# “Giros de Rua” - Revitalize physical and mental health in people with HIV through a psychosocial intervention program: a study protocol

**DOI:** 10.1192/j.eurpsy.2022.1941

**Published:** 2022-09-01

**Authors:** C. Laranjeira, A. Querido

**Affiliations:** Polytechnic of Leiria, School Of Health Sciences/ Citechcare, Leiria, Portugal

**Keywords:** mental health, PSYCHOSOCIAL REHABILITATION, Recovery, vulnerable population

## Abstract

**Introduction:**

Given the negative social representation attributed to mental illness, it is urgent to reconfigure its representation, not as an individual problem, but also as a collective. Several solutions have been indicated to improve the skills and quality of life (QoL) of these individuals. Among these, psychosocial rehabilitation programs stand out to keep individuals inserted in society reaching a level of independent functioning.

**Objectives:**

This study aims to: - assess the level of QoL, social support, treatment adherence, mental health status and mental health literacy of Portuguese people with HIV integrated into a community intervention program “InPulsar - Giros na Rua” program [which aims to contribute to the improvement of the socio-sanitary conditions of people who use psychoactive substances and to their social and therapeutic referral, as well as promoting risk reduction by intervening in a public space]; - improve mental health literacy levels, adherence to therapeutic regimen and QoL in individuals with HIV, after 12 months of implementation of a psychosocial rehabilitation program [biweekly sessions].

**Methods:**

We will perform a single-group pre-experimental study with pre- and post-intervention evaluation, supported by the participatory methodology in health.

**Results:**

This study has received ethical approval from the local IRB. Data collection will start in November 2021 and will be completed in November 2022.

**Conclusions:**

Through a multidisciplinary approach, this study will allow the development of health interventions articulated with psychosocial interventions based on various educational and behavioural strategies, promoting literacy and adherence to the therapeutic regimen.

**Disclosure:**

No significant relationships.

